# What we can and cannot learn from SARS-CoV-2 and animals in metagenomic samples from the Huanan market

**DOI:** 10.1093/ve/vead077

**Published:** 2023-12-29

**Authors:** Florence Débarre

**Affiliations:** Institute of Ecology and Environmental Sciences, Sorbonne Université, CNRS UMR 7618, UPEC, IRD, INRAE, Paris, France

**Keywords:** COVID-19 origin, Wuhan, zoonosis, forensic metagenomics

## Abstract

While the exact context of the emergence of SARS-CoV-2 remains uncertain, data accumulated since 2020 have provided an increasingly more precise picture of Wuhan’s Huanan Seafood Wholesale Market, to which the earliest clusters of human cases of Covid-19 were linked. After the market closed on January 1st 2020, teams from the Chinese Center for Disease Control and Prevention collected environmental samples, and sequenced them. Metagenomic sequencing data from these samples were shared in early 2023. These data confirmed that non-human animals susceptible to SARS-CoV-2 were present in the market before it closed, but also that these animals were located in the side of the market with most human cases, and in a corner with comparatively more SARS-CoV-2-positive environmental samples. The environmental samples were however collected after abundant human-to-human transmission had taken place in the market, precluding any identification of a non-human animal host. Jesse Bloom recently investigated associations between SARS-CoV-2 and non-human animals, concluding that the data failed to indicate whether non-human animals were infected by SARS-CoV-2, despite this being an already acknowledged limitation of the data. Here I explain why a correlation analysis could not confidently conclude which hosts(s) may have shed SARS-CoV-2 in the market, and I rebut the suggestion that such analyses had been encouraged. I show that Bloom’s investigation ignores the temporal and spatial structure of the data, which led to incorrect interpretations. Finally, I show that criteria put forward by Bloom to identify the host(s) that shed environmental SARS-CoV-2 would also exclude humans. Progress on the topic of SARS-CoV-2’s origin requires a clear distinction between scientific studies and news articles (mis)interpreting them.

## 1. Introduction

The question of the origin of the Covid-19 pandemic is inherently scientific, even though its broader political context and implications cannot be ignored (Gostin and Gronvall, 2023). Uncertainty remains regarding the exact conditions of SARS-CoV-2’s emergence and spillover to humans. The level of available details and the amount of data on the early days of Covid-19 are, however, remarkable compared to other emerging diseases, and in particular to SARS (Xu et al., 2004; Keusch et al., 2022).

In early 2020, a team from the Chinese Center for Disease Control and Prevention (CCDC) collected environmental samples in Wuhan’s Huanan Seafood Wholesale Market (hereafter ‘Huanan market’), to which cases of a new pneumonia were linked (Chen et al., 2020; Worobey, 2021). The market was sampled multiple times over several weeks, with various sampling strategies (World Health Organization, 2021; Liu et al., 2023b). Results from the first two sampling trips, which took place on January 1st and 12th 2020, were communicated to the press at the end of January 2020,^1^$^,$^2^ and were detailed in a report, which was not public at the time^3^ (Wu, 2020). When these first results were reported, the CCDC team had collected 585 samples, of which 33 had been found positive for the new coronavirus. The positive samples were in their vast majority (31/33) from the West side of the market, where wildlife was sold. The potential role of wildlife sold at the market was explicitly mentioned by CCDC (Wu, 2020; Tan et al., 2020).

The existence of metagenomic sequencing data related to the Huanan market environmental samples was mentioned in the report of the 2021 Joint World Health Organization (WHO)—China mission (World Health Organization, 2021, p.97). However, only maps of SARS-CoV-2 detection were presented, and raw data were not publicly shared.

The first results on the metagenomic content of the market samples were provided in a preprint by Liu et al. (2022) in early 2022. The corresponding raw data were not shared either at the time. The preprint did not describe in detail the contents of the environmental samples; humans were the only species named. A figure (Figure 4A in Liu et al. (2022)), however, indicated that other species besides humans had been detected in the data. In the absence of labels on the figure, of any description in the preprint’s main text, and of raw data, the identities of these species were unknown to the readers. In addition, the figure itself was criticized both for its form and its interpretation. It displayed Pearson correlation as a function of Spearman correlation, i.e. a correlation of correlations, and did not include error bars, therefore giving a false impression of certainty. The figure was used to conclude that humans were the source of SARS-CoV-2 in the Huanan market, a conclusion that could not be drawn from the analysis performed.

Multiple researchers publicly regretted the absence of raw data, and some, led by Jesse Bloom, even organized a petition to ask for the publication of these data.^4^

On January 30th 2023, a subset of the raw sequencing data became public on GISAID.^5^ The submission date displayed on GISAID was, however, in June 2022. The new data, therefore, appeared down in the database, and they apparently remained unseen for weeks. I first came across the data by chance on March 4th 2023, while looking for related information in the database. In the table of results on GISAID’s website, the records were reported as being 3-nucleotide long; the FASTA field was ‘AAA.’ Puzzled, and initially erroneously thinking that the newly found accessions were just placeholders awaiting data, I notified over the course of the next few days some colleagues of my discovery. These colleagues included Alex Crits-Christoph (on March 4th) and Jesse Bloom (on March 9th). After a brief email exchange with Jesse Bloom, I noticed the presence of FASTQ buttons on GISAID, indicating that raw data were in fact available. I communicated this new information to Alex Crits-Christoph and Jesse Bloom, together with the accession numbers of the data. Of these two, only Alex Crits-Christoph showed immediate interest in the data, downloaded them, and started analyzing them.

The dataset shared on GISAID at the time corresponded to 50 samples from the Huanan market, and data were available for 49 of them (Crits-Christoph et al., 2023a). The dataset only contained samples that had been tested positive for SARS-CoV-2.

Because previous work (Worobey et al., 2022a) had identified a specific market stall with an unusual number of samples reported as positive for SARS-CoV-2, our initial analysis focused on the five available samples from this stall. Worobey et al. (2022a) had made the direct prediction that SARS-CoV-2 susceptible wildlife may be present and detectable within the sequencing samples from this stall. The very first result we obtained with the data downloaded from GISAID was the detection of raccoon dog mitochondrial DNA (mtDNA) in the data from sample Q61. This species, shown to be susceptible to SARS-CoV-2 and able to transmit it (Freuling et al., 2020), was not named by Liu et al. (2022), and was not listed as a species present in the market in the 2021 WHO-China report (World Health Organization, 2021). A study published in the summer 2021 had identified them as animals sold in Wuhan markets (Xiao et al., 2021), but the results combined multiple markets and all the 31 months during which the study had been conducted, leading to the speculation that the animals may have been absent in the late Fall 2019. Finding genetic traces of these animals was, therefore, an important result. This initial result was later confirmed: raccoon dog reads were abundant across wildlife stall samples, and particularly so in a wildlife stall positive for SARS-CoV-2 (Crits-Christoph et al., 2023b).

In the early hours of March 11th 2023, the data could not be accessed on GISAID anymore (Crits-Christoph et al., 2023a, foreword), and would remain publicly inaccessible until March 29th 2023.

The discovery of the data and further initial results were communicated to the WHO’s Scientific Advisory Group for the Origins of Novel Pathogens (SAGO). Shortly after an online meeting with SAGO on March 14th, a journalist contacted one member of our group,^6^ thereby revealing to us that the news had leaked to the press. This first journalist only knew of generic points communicated to SAGO, and not of the context of the discovery of the data. At that time, we had only generated some figures that were presented to SAGO, but our analyses were not finalized, and we did not yet have a written report to share. We were not ready, nor willing, to have our results publicized. Another journalist aware of the news, however, went ahead, managing to convince some of us to be interviewed. Importantly, it was not even clear to some of those who were interviewed that the news would be published before our report was ready. The scoop was published on March 16th with a flashy title misrepresenting our results.^7^ I had explicitly and at multiple times declined to be interviewed by this journalist, and yet I ended up being quoted in a revised version of the article—the quote was taken, without consent, from an email asking for a correction that I sent two days after the article first came out. These anecdotes illustrate that scientists have no control over what journalists write; it is therefore essential to differentiate a scientific article from what journalists may write about it.

We published a report a few days later (Crits-Christoph et al., 2023a), accompanied by a foreword detailing the peculiar context in which the work was done. We explained why the results could not be reproduced, as data were no longer available. The report focused on the description of animals susceptible to SARS-CoV-2 in the market environmental sequences, including but not limited to raccoon dogs. This report contained a paragraph (pp. 13–14) preemptively listing issues that would be met if attempting to conduct a correlation analysis if the whole dataset ever became available.

Nine days later, Liu et al. published an updated version of their study on ChinaXiv (Liu et al., 2023a) and made their whole dataset available on public repositories (NGDC and SRA). There were now sequence data available for 172 samples (159 from the market, 3 from related warehouses, and 10 from sewage in the area). This new dataset notably now included samples tested negative for SARS-CoV-2. In their updated preprint, which was soon after accepted in Nature, Liu et al. (2023b) briefly described animal species detectable in the data. The Figure 4A of their preprint was updated after peer review into supplementary tables of tests of co-presence (Liu et al., 2023b, Supplementary Table 9) and into quantitative comparisons of animal contents in PCR-positive vs. PCR-negative samples (Liu et al., 2023b, Supplementary Table 7).

Finally, in April 2023, Bloom (2023a) posted a preprint analyzing the sequence data from Liu et al. (2023b), eventually published in Virus Evolution. In the article, Bloom (2023b) reproduced versions of Liu et al. (2022)’s Figure 4A, and concluded that the analysis did not allow for the identification of infected animals. While I agree with the conclusion—and more generally, with the fact that it is not possible to prove with these data that animals were infected, I would like to point out several important issues with Bloom’s article.

## 2. Results

### 2.1 Questioning the motivation for doing an analysis that we knew would fail

There are statistical errors, technical limitations, experimental imbalances, and conceptual failings behind the correlation analyses as described in Bloom (2023b), rendering them ultimately meaningless. Several conceptual issues were noted *a priori* (Crits-Christoph et al., 2023a); we further described them in our reply to Jesse Bloom’s preprint on bioRxiv,^8^ and in a recent preprint (Crits-Christoph et al., 2023b).

First, it is not possible to prove that non-human animals were infected, because the Huanan market samples were collected too late. This inescapable limitation was already identified by Jesse Bloom in 2022,^9^ and clearly stated in Bloom (2023b). (For simplicity, in the rest of the text, ‘animals’ means non-human animals.) There were a lot of human cases in the market, and most SARS-CoV-2 detected in the market are definitely of human origin. Given that it is impossible, with these data, to distinguish between viruses shed by humans and viruses shed by animals, any animal signal at the market scale was covered by human signal before the samples were collected. A correlation at the scale of the whole market cannot therefore identify infected animals, if there were any.

Even if the samples had been collected in the very early days of SARS-CoV-2’s presence in the Huanan market, a correlation analysis could have failed to identify the source species. Such an analysis indeed implicitly assumes that most animals of a species were infected. Different stalls selling the same species did not necessarily have the same suppliers. This was for instance the case for bamboo rats, which came from origins as different as Hubei, Guangxi, Hunan, and Yunnan provinces (World Health Organization, 2021, annexes pp.189–191). If only a fraction of animals of a given species were infected, a correlation analysis may fail to identify the species as the source. We observe this phenomenon in the market data with another virus, H3N2 influenza virus (Crits-Christoph et al., 2023b). This human virus is present in a single (non-wildlife) stall in the market, probably because only a limited number of humans had this flu virus in Fall 2019 in the market. A market-wide correlation with human reads is not significant, because many other humans in the market were not infected (log proportion of total reads, cor = 0.076 (95 per cent CI: −0.081, 0.23), *p* = 0.34; data: Crits-Christoph et al. (2023b)). This shows that even if the samples had been collected much earlier, before the many human infections that followed, an animal source of SARS-CoV-2 would still probably not have been identifiable with a correlation analysis.

Then why conduct a market-wide correlation analysis in the first place, when it is acknowledged that the samples were collected comparatively too late for such an analysis to be insightful? A motivation is given by Bloom (2023b): The absence of labels in Liu et al. (2022)’s Figure 4A had been widely noted (Bloom (2023b) cited three news articles by Jon Cohen to back up the claim), but neither Crits-Christoph et al. (2023a) nor Liu et al. (2023b) had ‘addressed this omission’; Bloom (2023b) study is here ‘[t]o remedy this omission.’

This is a misrepresentation of the reasons why the absence of labels was noted. None of the scientists quoted by Jon Cohen, nor even Jon Cohen himself (personal communications and contemporary statements^10^), meant that it would be worth reproducing Figure 4A. The figure was only used as evidence that species were undescribed in Liu et al. (2022). This omission was addressed by Crits-Christoph et al. (2023a) and Liu et al. (2023b), who described and quantified animals reads detectable in the metagenomic sequence data. There is just no good rationale for conducting a market-wide univariate correlation analysis to identify a potential infected non-human animal with these data.

In his article, Bloom (2023b) emphasized that his results were inconsistent with ‘media articles that emphasized the co-mingling of raccoon dog and viral material (Wu 2023; Mueller 2023).’^11^ It seems that countering these media articles was an important motivation for Bloom’s study. However, the meaning of co-mingling in these articles is mischaracterized by Bloom: it was not meant at the scale of the whole market, but in a specific stall. Both Wu’s and Mueller’s articles emphasized the fact that raccoon dog genetic material and SARS-CoV-2 had been found together in a sample (Q61). A quote in Mueller’s article clarified that the data ‘can’t prove definitely there was an infected animal at that stall,’ and Mueller specified that ‘even if a raccoon dog had been infected, it would not be clear that the animal had spread the virus to people’—i.e. statements matching Bloom (2023b)s conclusions. Wu used the word ‘co-mingled’ in ‘Finding the genetic material of virus and mammal so closely co-mingled–enough to be extracted out of a single swab–isn’t perfect proof,’ and this sentence was clearly again about sample Q61. Neither of these articles was about market-wide co-presence, so it is incorrect to suggest that a market-wide analysis contradicts Mueller’s and Wu’s articles.

Bloom’s study itself was misinterpreted by commenters and misrepresented in the media, notably as evidence that the raccoon dogs were not infected (instead of as absence of evidence that they were). This and confusion between our report and news articles led to particularly vitriolic attacks on our own work ( e.g. ‘Why Does Bad Science on Covid’s Origin Get Hyped?’ in a New York Times newsletter.^12^).

Even if there had been good reasons to conduct such a correlation analysis at the scale of the whole market, there are substantial issues with the way it is done in Bloom (2023b).

### 2.2 Temporal and spatial structures in the data cannot be omitted

The unbalanced sequencing design in the data shared by Liu et al. (2023b, Extended Data Table 2) precludes any market-wide analysis. The Huanan market was sampled multiple times; all samples were tested by PCR for SARS-CoV-2, and only a fraction were subjected to metagenomic sequencing. The fraction and characteristics of the samples that were sequenced varied over time. The first collection trip, on January 1st 2020, focused on stalls with human cases, and only samples positive by PCR were then sequenced. The second collection trip, on January 12th 2020, focused on wildlife stalls; an equal number of samples were collected from each stall, and all were sequenced irrespective of PCR positivity. For later trips, only a fraction of samples were sequenced, both from PCR-positive and PCR-negative samples. These later trips also included samples from outside the market (including warehouses related to the market; sewage around the market). The proportion of SARS-CoV-2 positive samples among the sequenced samples therefore widely varied over time, as did the composition of animal species (wildlife stalls had different species than other stalls in the market). It is therefore incorrect to present correlation analyses mixing up data from all sampling trips, as is done in most of Bloom (2023b)’s article. Mixing up these data leads to instances of Simpson’s paradox: some correlations substantially change once the time structure is taken into account (Crits-Christoph et al., 2023b, Figure S4).

**Figure 1.**
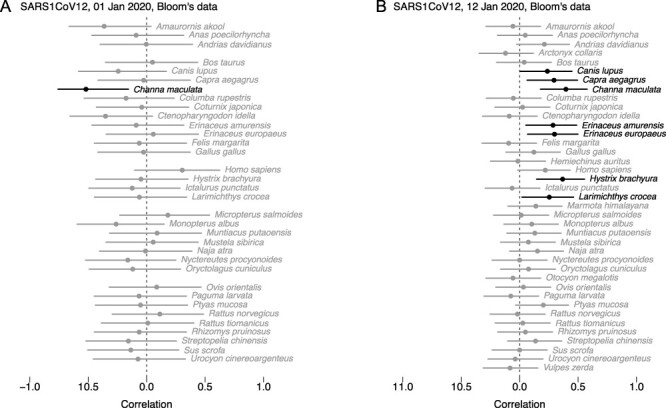
Pearson correlations on log proportion of total reads between SARS-CoV-2 and chordate species; points in black correspond to statistically significant values (*p* < 0.05). (A) Samples collected on January 1st 2020; sequenced samples were all PCR-positive (*n* = 25). (B) Samples collected on January 12th 2020; all samples were sequenced (*n* = 70). Data: Bloom (2023b).

One subset of the data can be considered as balanced. All samples collected on January 12th 2020 were sequenced, irrespective of SARS-CoV-2 PCR positivity; ten samples were collected in each of the seven stalls identified as selling wildlife. Because these samples were collected late, however, SARS-CoV-2 reads are rare: six samples are positive, with five in the same stall (‘stall A’ in Crits-Christoph et al. (2023b)). There are 1, 2, 2, 5, and 7 reads in the positive samples from stall A, and 5 reads from the positive samples from stall B; 0 reads in the 64 other samples collected on January 12th 2020. It does not make much sense to compute a correlation with such data. However, if we do it, humans (*Homo sapiens*) are not significantly associated with SARS-CoV-2 (log proportions of total reads; cor = 0.22 (95 per cent CI: −0.017, 0.43), *p* = 0.068; data: Bloom (2023b) from inside the market; see Figure 1B and Supplementary Table S1). Raccoon dogs (*Nyctereutes procyonoides*) are not significantly associated either (cor = −0.00055 (95 per cent CI: −0.24, 0.23), *p* = 1). A correlation analysis on this subset of data therefore does not exclude raccoon dogs as potential source, and does not identify humans as potential source.

Consideration of the spatial and temporal structure of the samples helps confirm their positive or negative status. In his article, Bloom (2023b) emphasized the low number of reads in sample Q61 (‘1 of  200,000,000 reads’), and stressed the lack of significant difference between 1 read in sample Q61 vs. 0 reads in sample E-10-29-2. Bloom’s analysis however neglected the context in which the samples were collected. When taking into account the spatial and temporal structure of the data, Q61 is clearly positive. The maximum number of SARS-CoV-2 reads in the other samples collected on January 12th 2020 is 7, i.e. also very low. Out of the six positive samples collected on that date, five including Q61 come from the very same stall, and three of them were positive by PCR (Crits-Christoph et al., 2023b). Likewise, sample E-10-29-2 is clearly negative. Only one out of 27 samples from inside the market collected on the same day (February 20th 2020) was positive by PCR, and none of the 52 samples from inside the market collected after that date was tested positive by PCR or by sequencing. In addition, no other sample in the stall and adjacent locations was ever tested positive either. Even though a chi-squared test on the proportions of reads in Q61 vs. E-10-29-2 is non-significant, their spatial and temporal contexts confirm the results.

### 2.3 Criteria excluding raccoon dogs as hosts also exclude humans

Bloom (2023b) argued that only one sample (Q61) had a substantial proportion of raccoon dog reads and any presence of SARS-CoV-2, ‘substantial’ being defined as at least 20 per cent of chordate reads. In the previous section, I explained why this sample is clearly SARS-CoV-2-positive in spite of its very low number of reads; here I focus on the choice of criteria to call a proportion of reads of a given species ‘substantial.’ After these criteria had been pointed out, Jesse Bloom argued that the threshold had been chosen simply to produce a table of reasonable length; yet the argument remained in the abstract. Twenty per cent of chordate reads is a very stringent threshold. If we apply the same threshold to human reads on Bloom’s data, then among the 33 samples from inside the market with SARS-CoV-2 reads, only eight have at least 20 per cent of chordate reads belonging to *Homo sapiens* (Supplementary Table S2). Among these eight samples, five are from stalls with human cases on the map of the Joint China-WHO mission (World Health Organization, 2021), two are in stalls with human cases on another map produced by CCDC (The BMJ, 2021), and one is next to stalls with human cases on this other map. Twenty-five other samples from inside the market contain SARS-CoV-2 reads but less than 20 per cent human reads among chordates reads. Yet SARS-CoV-2 in at least one of these is almost certainly of human origin: sample B5 comes from a stall with two to three cases; SARS-CoV-2 was so abundant in the sample that an almost full sequence could be assembled, and live virus could be isolated; its sequence matches the reference sequence. In spite of this clear association with a human case, the percentage of human reads in this sample is only 0.12 per cent.

Market-wide correlations do not identify humans as hosts either. In the text of the article, Bloom highlights the negative correlation between raccoon dog reads and SARS-CoV-2 reads. As already explained, correlations on all samples are plagued by the fact that the sampling and sequencing strategies changed over time, and Simpson’s paradox is at play. Focusing on a homogeneous subset of the data, collected on January 12th, I also already pointed out that correlations were not statistically significant for raccoon dogs nor for humans. We can additionally consider the January 1st data: this first trip focused on stalls with human cases; only samples positive by PCR were sequenced. Even on this subset of data for which humans were the likely source of potentially all SARS-CoV-2 viruses, the correlation is not significant for humans (cor = 0.3 (95 per cent CI: −0.1, 0.62), *p* = 0.14; data: Bloom (2023b); Figure 1A). (Note that a labeling error makes humans appear more correlated to SARS-CoV-2 than they really are in the bottom-left panel of Bloom (2023b)’s Figure 4.) Humans stand out even less if a larger set of species is included in the reference dataset (Supplementary Figure S1). Finally, Bloom (2023b) highlighted a high positive correlation between SARS-CoV-2 and a fish species, largemouth bass (*Micropterus salmoides*). This non-sensical result by itself illustrated that market-wide correlations cannot identify hosts. We however need to note that the result was largely driven by the exclusion of two samples, F13 and F54. Once these metagenomic samples are added back, the correlation is much weaker and non-significant (see Supplementary Table S1).

## 3. Discussion

There are major scientific issues with the original results presented in Bloom (2023b). The market-wide correlations on all samples from all locations and at all collection dates lump together data that cannot be analyzed together, because the samples were collected with different purposes and were sequenced with different rationales. The correlation values are therefore uninterpretable, and many of these values are affected by an instance of Simpson’s paradox: they change once structure in the data is taken into account. Many of the values put forward are actually not statistically significant. The criteria that are applied to non-human animals are so stringent that they would exclude humans as a source of SARS-CoV-2 in many samples. Finally, the discussion of the SARS-CoV-2-positive or -negative nature of some samples ignores the spatial and temporal structure of the data.

There is also a major issue with the presentation of the motivation for the study. Contrary to what is written in Bloom (2023b), and as previously highlighted in a reply to the first version of the preprint of this work,^13^ it was not suggested that it would be valuable to reproduce Liu et al. (2022)’s Figure 4A. It was obvious before doing it (Crits-Christoph et al., 2023a) that such an approach would not identify an animal host—not to mention the odd choice of plotting a correlation of correlations. In addition, Bloom (2023b) does not contradict news reports on ‘co-mingling’ of SARS-CoV-2 and raccoon dogs, because these articles were focused on one *stall*, which was clearly positive for SARS-CoV-2, while Bloom’s study is at the scale of the whole market. In the news articles criticized by Bloom, none of the interviewed collaborators of Crits-Christoph et al. (2023a) mentioned co-presence of SARS-CoV-2 and raccoon dogs at the scale of the whole market.

The metagenomic sequence data from the Huanan market are observational: by nature, they cannot prove any hypothesis. However, they are not uninformative: with them, several key predictions of the zoonotic hypothesis of SARS-CoV-2’s origin could be tested. Before the contents of these data were revealed, falsifiable predictions had indeed been made:

*i) If the origin is at the market, then lineage A should be in the market.* Early SARS-CoV-2 sequences were grouped into two lineages, A and B, separated by two characteristic mutations. Initially, however, lineage A had not been associated with the market. Absence of evidence is not evidence of absence, so this absence of detection did not disprove a market origin, but it was nevertheless seen as a serious limitation for a single Huanan market-origin scenario (Zhang et al., 2020; Bloom, 2021). Worobey et al. (2022b) had predicted that, given the location of the two earliest publicly known lineage A cases, lineage A had to be in the market. Liu et al. (2022) revealed that lineage A was indeed in the market, found in sample A20, which independently validated the prediction. The detection of lineage A in the market is a major finding, because it implies that the most recent common ancestor of SARS-CoV-2 was also related to the market (Babarlelephant et al., 2022).

*ii) If the virus comes from infected animals, then there should be human infections and positive environmental samples near stalls selling animals.* Most cases are from the West side of the market, and it was recognized as early as January 2020 that this was where wildlife stalls were located. The details published by Liu et al. (2023b) on the numbers of samples collected in each stall, and the associated PCR and sequencing results, allowed the identification of a positivity hotspot in the market, controlling for sampling effort (Crits-Christoph et al., 2023b). This result rebutted the claim that more positive samples had been found in the South-West corner of the West side because more samples had been collected there.

*iii) If the virus comes from infected animals, then there should exist stalls in which the genetic materials of SARS-CoV-2 and of susceptible animals are detectable together.* This prediction was also confirmed by the metagenomic sequence data from the Huanan market. Strikingly, the stall (stall A) in which susceptible animals and SARS-CoV-2 are detected together happens to be next to the positivity hotspot.

Predictions *ii)* and *iii)* could have been disproved by the Huanan market data. Positive samples or cases could have been predominantly in the East side of the market. Susceptible animals could have been totally disjoint from SARS-CoV-2, as is the case a few streets up stall A, where wildlife was sold but no SARS-CoV-2 was detected, even though the stalls were heavily sampled (Liu et al., 2023b; Crits-Christoph et al., 2023b).

An animal origin does not imply that all SARS-CoV-2 in the market was shed by (non-human) animals, nor that all humans were infected by animals. Bloom (2023b) cited an article by Courtier-Orgogozo and de Ribera (2022) as an alternative interpretation of Liu et al. (2022)’s data. This article proposes that the toilets or a mahjong room, both in the South-West corner of the West side of the market, were locations where human-to-human transmission took place. It is important to note that these suggestions are not incompatible with an animal origin—quite the contrary actually. Most SARS-CoV-2 detected in the market was shed by humans, and most humans were infected by other humans. A fraction of these infections can have taken place in places where people gathered, without precluding in any way the fact that a couple of people may have been initially infected by animals. The news article^14^ at the origin of the suggestion that toilets may have played a role in transmission is actually even a good argument for an origin linked to the wildlife trade in the market, when the relevant quote is read in full (emphasis added): ‘Looking back, Ms [W.] thinks she might have been infected via the toilet she *shared with the wild meat sellers* and others on the market’s west side.’

Proving that non-human animals were infected would not prove that SARS-CoV-2 first entered the Huanan market inside of non-human animals. It could still be argued, as is done in Liu et al. (2023b) and Bloom (2023b), that the animals could have been infected by humans. Likewise, it can be argued that the detection of SARS-CoV-2 on a cage could be from a human coughing on the cage, and not from an animal inside of it. At some point however, making sense of all the available data—including a feature not discussed yet, the distribution of early cases around the market (Worobey et al., 2022a)—becomes unreasonably difficult under a lab leak scenario. It then becomes necessary to invoke deliberately hidden data, as is done in Bloom (2023b) with his reference to Hvistendahl and Mueller’s news article.^15^ This reference does not only amount to suggesting that the Chinese government is withholding data, but also that Chinese scientists are hiding or manipulating such data in their own articles.

To conclude, the metagenomic data from the Huanan market cannot by nature prove that a non-human animal was infected, and it was known before even conducting it that a market-wide correlation analysis on these data would not prove it either. Crits-Christoph et al. (2023a) did not pretend it would be the case. In addition, Bloom (2023b)’s analysis had substantial scientific issues. Finally, Bloom (2023b)’s article listed ‘lab leak’ as a keyword, even though the Huanan market data do not directly inform this other hypothesis for the origin of SARS-CoV-2. Whether or not keeping the discussion alive was the intention of the article, this was its outcome in the media. Maybe such aims are better served by opinion pieces, rather than by conducting a study doomed to fail in order to argue that nothing can be concluded from a dataset. The metagenomic sequencing data from Huanan market samples are among the most precious and insightful datasets related to the origin of the pandemic shared by Chinese researchers. Even though they were shared late, we can be grateful that these data exist and are now publicly available.

## 4. Methods

I downloaded analysis results by Bloom (2023b) (https://github.com/jbloom/Huanan_market_samples) and by Crits-Christoph et al. (2023b) (https://github.com/sars-cov-2-origins/huanan-market-environment). I excluded samples from outside the market: sewage from surrounding areas, and warehouses, because SARS-CoV-2 in such samples can have been deposited after the closure of the Huanan market. I also excluded samples corresponding to amplicon-based SARS-CoV-2 whole genome sequencing. I kept all samples from the Huanan market and subjected to metagenomic sequencing (Source: METAGENOMIC and Strategy: RNA-Seq in Liu et al. (2023b)’s metadata).

The cor.test function in R was used to compute correlations, confidence intervals, *p* values.

## Supplementary Material

vead077_Supp

## Data Availability

R scripts to reproduce the results are available at https://github.com/flodebarre/Huanan-env_Bloom-reply, preserved on Zenodo at https://doi.org/10.5281/zenodo.10278323.

## Footnotes

1 References that are not scientific articles or reports are presented as footnotes.

2 e.g. http://www.xinhuanet.com/english/2020-01/27/c_138735677.htm, https://news.cgtn.com/news/2020-01-27/Experts-confirm-Wuhan-seafood-market-was-source-of-novel-coronavirus-NAHPUtsPgA/index.html.

3 The report was leaked later in 2020; https://www.epochtimes.com/gb/20/5/31/n12150755.htm.

4 cf. Twitter discussion, February 27th 2022; https://x.com/jbloom_lab/status/1497743165226303489.

5 The date was provided by GISAID https://www.science.org/content/article/it-s-inexcusable-who-blasts-china-not-disclosing-potential-data-covid-19-s-origin and this date range confirmed by comparing metadata downloads from various time points.

6 Later publicly confirmed by a tweet; https://archive.ph/2MDB9.

7 Katherine J. Wu, *The Strongest Evidence Yet That an Animal Started the Pandemic*; https://www.theatlantic.com/science/archive/2023/03/covid-origins-research-raccoon-dogs-wuhan-market-lab-leak/673390/.

8 Reply by Florence Débarre &; Alex Crits-Christoph posted on bioRxiv; http://disq.us/p/2u34bgh.

9 As noted publicly in a tweet; https://x.com/jbloom_lab/status/1497627222206664706.

10 e.g. Twitter thread by Andrew Rambaut, 2022-02-26; https://archive.ph/tuhgM.

11 Wu’s article is referenced in footnote 7; Benjamin Mueller, *New Data Links Pandemic’s Origins to Raccoon Dogs at Wuhan Market*  https://www.nytimes.com/2023/03/16/science/covid-wuhan-market-raccoon-dogs-lab-leak.html.

12 David Wallace-Wells; https://www.nytimes.com/2023/05/03/opinion/covid-origin-science.html.

13 See footnote 8.

14 Jeremy Page, Wenxin Fan and Natasha Khan, *How It All Started: China’s Early Coronavirus Missteps*; https://www.wsj.com/articles/how-it-all-started-chinas-early-coronavirus-missteps-11583508932.

15 Mara Hvistendahl and Benjamin Mueller, *Chinese Censorship Is Quietly Rewriting the Covid-19 Story*; https://www.nytimes.com/2023/04/23/world/europe/chinese-censorship-covid.html. See https://github.com/flodebarre/Shen-etal_2020 for a critical re-analysis of one of their claims.
