# Supplementary material for: What we can and cannot learn from SARS-CoV-2 and animals in metagenomic samples from the Huanan market
Source: Virus Evol. 2023 Dec 29;10(1):vead077. doi: 10.1093/ve/vead077 (PMC10868546; doi:10.1093/ve/vead077)
Supplement: vead077_Supp [file vead077_supp.zip › tableS1.docx]

| **Virus** | **Host** | **Dates** | **Dataset** | **Result** |
| --- | --- | --- | --- | --- |
| SARS-CoV-2 | Human | Jan 12 | ACC | cor = $0.2$ (95% CI: $-0.036$, $0.42$), $p$ = $0.095$ |
|  |  |  | JB | cor = $0.22$ (95% CI: $-0.017$, $0.43$), $p$ = $0.068$ |
| SARS-CoV-2 | Raccoon dog | Jan 12 | ACC | cor = $-0.042$ (95% CI: $-0.27$, $0.19$), $p$ = $0.73$ |
|  |  |  | JB | cor = $-0.00055$ (95% CI: $-0.24$, $0.23$), $p$ = $1$ |
| SARS-CoV-2 | Human | Jan 01 | ACC | cor = $0.18$ (95% CI: $-0.23$, $0.54$), $p$ = $0.38$ |
|  |  |  | JB | cor = $0.3$ (95% CI: $-0.1$, $0.62$), $p$ = $0.14$ |
|  |  |  | JB2 | cor = $0.33$ (95% CI: $-0.09$, $0.66$), $p$ = $0.12$ |
| SARS-CoV-2 | Spotted bass | Jan 01 | ACC | cor = $-0.016$ (95% CI: $-0.41$, $0.38$), $p$ = $0.94$ |
|  | Largemouth bass |  | JB | cor = $0.18$ (95% CI: $-0.23$, $0.54$), $p$ = $0.39$ |
|  |  |  | JB2 | cor = $0.41$ (95% CI: $0.0024$, $0.71$), $p$ = $0.049$ |
| H3N2 | Human | all | ACC | cor = $0.076$ (95% CI: $-0.081$, $0.23$), $p$ = $0.34$ |
