# Supplementary material for: What we can and cannot learn from SARS-CoV-2 and animals in metagenomic samples from the Huanan market
Source: Virus Evol. 2023 Dec 29;10(1):vead077. doi: 10.1093/ve/vead077 (PMC10868546; doi:10.1093/ve/vead077)
Supplement: vead077_Supp [file vead077_supp.zip › tableS2.docx]

Table S2: Proportion of human reads among chordate reads, in samples with and without SARS-CoV-2 reads; data from Bloom (2023a). A machine-readable csv source is available on the projet’s repository.

| Sample ID | Lab code | Proportion human reads | Number SARS-CoV-2 reads |
| --- | --- | --- | --- |
| Env_0346 | F46 | 0.9051 | 241 |
| Env_0398 | F98 | 0.7665 | 330 |
| Env_0313 | F13 | 0.6386 | 420581 |
| Env_0354 | F54 | 0.6306 | 97675 |
| Env_0015 | A15 | 0.5824 | 200 |
| Env_0018 | A18 | 0.4421 | 226 |
| Env_0213 | D32 | 0.4235 | 326 |
| Env_0138 | B17 | 0.3226 | 406 |
| Env_0275 | E61 | 0.1389 | 57 |
| Env_0063 | A63 | 0.1361 | 65 |
| Env_0101 | A101 | 0.1317 | 67 |
| Env_0221 | E7 | 0.1223 | 126 |
| Env_0820 | WWS-2#-2 | 0.1158 | 4 |
| Env_0583 | Q68 | 0.0719 | 6 |
| Env_0552 | Q37 | 0.0678 | 5 |
| Env_0087 | A87 | 0.0638 | 136 |
| Env_0719 | 8-25-M1 | 0.0453 | 24 |
| Env_0088 | A88 | 0.0351 | 18 |
| Env_0002 | A2 | 0.0313 | 66 |
| Env_0020 | A20 | 0.0227 | 55 |
| Env_0579 | Q64 | 0.0218 | 5 |
| Env_0061 | A61 | 0.0185 | 334 |
| Env_0584 | Q69 | 0.0110 | 2 |
| Env_0096 | A96 | 0.0091 | 4 |
| Env_0055 | A55 | 0.0087 | 176 |
| Env_0400 | F100 | 0.0067 | 7202 |
| Env_0585 | Q70 | 0.0032 | 2 |
| Env_0090 | A90 | 0.0013 | 22 |
| Env_0126 | B5 | 0.0012 | 1460 |
| Env_0262 | E48 | 0.0005 | 10 |
| Env_0576 | Q61 | 0.0004 | 1 |
| Env_0014 | A14 | 0.0003 | 4 |
| Env_0033 | A33 | 0.0001 | 35 |
| Env_0759 | Outside-5 | 0.9937 | 0 |
| Env_0751 | WS-4-1 | 0.9757 | 0 |
| Env_0748 | WS-2-3 | 0.9741 | 0 |
| Env_0842 | C12 | 0.9643 | 0 |
| Env_0749 | WS-3-1 | 0.9620 | 0 |
| Env_0847 | C17 | 0.9444 | 0 |
| Env_0746 | WS-2-1 | 0.8885 | 0 |
| Env_0753 | 8-25-D1 | 0.8834 | 0 |
| Env_0546 | HJ200031-20200112-1 | 0.8548 | 0 |
| Env_0619 | 10-31-abv2 | 0.8455 | 0 |
| Env_0617 | 10-31-In2 | 0.7497 | 0 |
| Env_0686 | 1-29-8 | 0.7097 | 0 |
| Env_0742 | WS-1-1 | 0.6813 | 0 |
| Env_0642 | 1-27-11 | 0.5636 | 0 |
| Env_0815 | EWS-3#-2 | 0.4886 | 0 |
| Env_0720 | 8-25-M2 | 0.4006 | 0 |
| Env_0620 | 06-29-abv1 | 0.3923 | 0 |
| Env_0643 | 1-27-12 | 0.2912 | 0 |
| Env_0756 | 8-25-Long | 0.2857 | 0 |
| Env_0893 | CCDC-744 | 0.2778 | 0 |
| Env_0818 | WWS-1#-3 | 0.2529 | 0 |
| Env_0817 | WWS-1#-2 | 0.2299 | 0 |
| Env_0839 | C9 | 0.2222 | 0 |
| Env_0697 | 1-29-19 | 0.2216 | 0 |
| Env_0830 | wws-1#-0 | 0.1949 | 0 |
| Env_0816 | WWS-1# | 0.1938 | 0 |
| Env_0909 | CCDC-760 | 0.1408 | 0 |
| Env_0714 | 8-25-BX | 0.1364 | 0 |
| Env_0754 | 8-25-D2 | 0.1315 | 0 |
| Env_0547 | HJ200032-20200112-1 | 0.1306 | 0 |
| Env_0856 | E-10-29-2 | 0.1250 | 0 |
| Env_0516 | HJ200001-20200112-1 | 0.0954 | 0 |
| Env_0889 | 629-L-1 | 0.0949 | 0 |
| Env_0821 | WWS-2#-3 | 0.0874 | 0 |
| Env_0549 | HJ200034-20200112-1 | 0.0860 | 0 |
| Env_0885 | 629-11-L | 0.0742 | 0 |
| Env_0548 | HJ200033-20200112-1 | 0.0690 | 0 |
| Env_0701 | 1-29-23 | 0.0639 | 0 |
| Env_0526 | HJ200011-20200112-1 | 0.0635 | 0 |
| Env_0555 | HJ200040-20200112-1 | 0.0633 | 0 |
| Env_0875 | 629-1-L1 | 0.0632 | 0 |
| Env_0525 | HJ200010-20200112-1 | 0.0611 | 0 |
| Env_0519 | HJ200004-20200112-1 | 0.0594 | 0 |
| Env_0806 | W-8-25-D1 | 0.0543 | 0 |
| Env_0550 | HJ200035-20200112-1 | 0.0507 | 0 |
| Env_0554 | HJ200039-20200112-1 | 0.0471 | 0 |
| Env_0535 | HJ200020-20200112-1 | 0.0467 | 0 |
| Env_0551 | HJ200036-20200112-1 | 0.0442 | 0 |
| Env_0536 | HJ200021-20200112-1 | 0.0442 | 0 |
| Env_0540 | HJ200025-20200112-1 | 0.0423 | 0 |
| Env_0553 | HJ200038-20200112-1 | 0.0414 | 0 |
| Env_0558 | HJ200043-20200112-1 | 0.0412 | 0 |
| Env_0712 | 7-26-DH | 0.0366 | 0 |
| Env_0907 | CCDC-758 | 0.0345 | 0 |
| Env_0882 | 629-8-L7 | 0.0340 | 0 |
| Env_0566 | HJ200051-20200112-1 | 0.0338 | 0 |
| Env_0674 | 1-27-52 | 0.0333 | 0 |
| Env_0537 | HJ200022-20200112-1 | 0.0330 | 0 |
| Env_0578 | HJ200063-20200112-1 | 0.0295 | 0 |
| Env_0705 | 7-26-BX | 0.0286 | 0 |
| Env_0813 | EWS-2#-2 | 0.0278 | 0 |
| Env_0533 | HJ200018-20200112-1 | 0.0258 | 0 |
| Env_0562 | HJ200047-20200112-1 | 0.0228 | 0 |
| Env_0545 | HJ200030-20200112-1 | 0.0208 | 0 |
| Env_0518 | HJ200003-20200112-1 | 0.0203 | 0 |
| Env_0541 | HJ200026-20200112-1 | 0.0180 | 0 |
| Env_0524 | HJ200009-20200112-1 | 0.0178 | 0 |
| Env_0523 | HJ200008-20200112-1 | 0.0171 | 0 |
| Env_0809 | W-8-25-L2 | 0.0163 | 0 |
| Env_0707 | 7-26-PSW | 0.0151 | 0 |
| Env_0887 | 629-13-L | 0.0133 | 0 |
| Env_0879 | 629-5-L4 | 0.0131 | 0 |
| Env_0528 | HJ200013-20200112-1 | 0.0128 | 0 |
| Env_0715 | 8-25-D_Env_0715 | 0.0119 | 0 |
| Env_0580 | HJ200065-20200112-1 | 0.0112 | 0 |
| Env_0532 | HJ200017-20200112-1 | 0.0102 | 0 |
| Env_0531 | HJ200016-20200112-1 | 0.0092 | 0 |
| Env_0575 | HJ200060-20200112-1 | 0.0086 | 0 |
| Env_0716 | 8-25-CK | 0.0082 | 0 |
| Env_0807 | W-8-25-D2 | 0.0080 | 0 |
| Env_0568 | HJ200053-20200112-1 | 0.0076 | 0 |
| Env_0560 | HJ200045-20200112-1 | 0.0073 | 0 |
| Env_0572 | HJ200057-20200112-1 | 0.0071 | 0 |
| Env_0657 | 1-27-28 | 0.0069 | 0 |
| Env_0544 | HJ200029-20200112-1 | 0.0067 | 0 |
| Env_0574 | HJ200059-20200112-1 | 0.0064 | 0 |
| Env_0542 | HJ200027-20200112-1 | 0.0061 | 0 |
| Env_0527 | HJ200012-20200112-1 | 0.0060 | 0 |
| Env_0543 | HJ200028-20200112-1 | 0.0055 | 0 |
| Env_0538 | HJ200023-20200112-1 | 0.0055 | 0 |
| Env_0534 | HJ200019-20200112-1 | 0.0054 | 0 |
| Env_0808 | W-8-25-L | 0.0051 | 0 |
| Env_0581 | HJ200066-20200112-1 | 0.0051 | 0 |
| Env_0570 | HJ200055-20200112-1 | 0.0050 | 0 |
| Env_0559 | HJ200044-20200112-1 | 0.0048 | 0 |
| Env_0708 | 7-26-PSN | 0.0046 | 0 |
| Env_0556 | HJ200041-20200112-1 | 0.0046 | 0 |
| Env_0564 | HJ200049-20200112-1 | 0.0045 | 0 |
| Env_0520 | HJ200005-20200112-1 | 0.0041 | 0 |
| Env_0829 | w-zong-1 | 0.0040 | 0 |
| Env_0530 | HJ200015-20200112-1 | 0.0040 | 0 |
| Env_0561 | HJ200046-20200112-1 | 0.0037 | 0 |
| Env_0895 | CCDC-746 | 0.0036 | 0 |
| Env_0577 | HJ200062-20200112-1 | 0.0035 | 0 |
| Env_0539 | HJ200024-20200112-1 | 0.0031 | 0 |
| Env_0529 | HJ200014-20200112-1 | 0.0031 | 0 |
| Env_0877 | 629-3-C | 0.0027 | 0 |
| Env_0569 | HJ200054-20200112-1 | 0.0026 | 0 |
| Env_0522 | HJ200007-20200112-1 | 0.0024 | 0 |
| Env_0521 | HJ200006-20200112-1 | 0.0020 | 0 |
| Env_0563 | HJ200048-20200112-1 | 0.0016 | 0 |
| Env_0571 | HJ200056-20200112-1 | 0.0012 | 0 |
| Env_0582 | HJ200067-20200112-1 | 0.0008 | 0 |
| Env_0567 | HJ200052-20200112-1 | 0.0007 | 0 |
| Env_0565 | HJ200050-20200112-1 | 0.0004 | 0 |
| Env_0573 | HJ200058-20200112-1 | 0.0000 | 0 |
| Env_0517 | HJ200002-20200112-1 | 0.0000 | 0 |
| Env_0557 | HJ200042-20200112-1 | 0.0000 | 0 |
| Env_0660 | 1-27-33 | 0.0000 | 0 |
| Env_0682 | 1-29-4 | 0.0000 | 0 |
| Env_0717 | 8-25-D_Env_0717 | 0.0000 | 0 |
| Env_0828 | w-6-29-33 | 0.0000 | 0 |
| Env_0858 | E-A-7-1 | 0.0000 | 0 |
| Env_0873 | CCDC-454 | 0.0000 | 0 |
| Env_0664 | 1-27-37 | NA | 0 |
| Env_0752 | WS-4-2 | NA | 0 |
|  |  |  |  |
